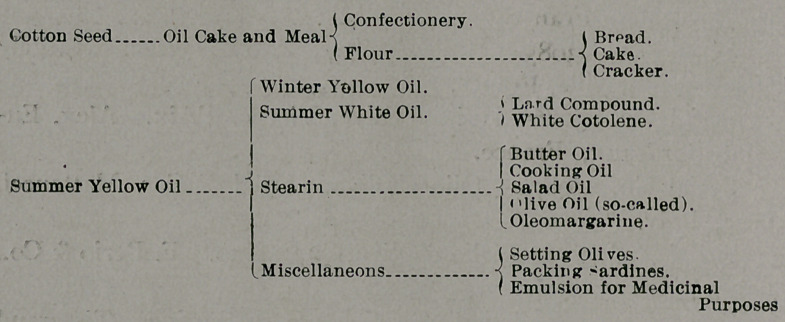# Etiology, Pathology and Treatment of Pellagra

**Published:** 1911-09

**Authors:** George C. Mizell

**Affiliations:** Atlanta, Ga.


Journal-Record of Medicine

Successor to Atlanta Medical and Surgical Journal, Established 1855, and Southern Medical Record, Established 1870.

OWNED BY THE ATLANTA MEDICAL JOURNAL CO.

Published Monthly

*Official Organ Fulton County Medical Society, State Examining Board, Presbyterian Hospital, Atlanta, Birmingham and Atlantic Railroad Surgeons' Association, Chattahoochee Valley Medical and Surgical Association, Etc.*

EDGAR G. BALLENGER., M. D., Editor.
BERNARD WOLFF, M. D., Supervising Editor.
A. W. STIRLING, M. D., C. M., D. P. H., J. S. HURT, B. Ph., M. D.
GEO. M. NILES, M. D., W. J. LOVE, M. D., (Ala.); Associate Editors.
E. W. ALLEN, Business Manager.

COLLABORATORS

Dr. W. F. WESTMORLAND, General Surgery.
F. W. McRAE, M. D., Abdominal Surgery.
H. F. HARRIS, M. D., Pathology and Bacteriology.
MICHAEL HOKE, M. D., Orthopedic Surgery.
CYRUS W. STRICKLER, M. D., Legal Medicine and Medical Legislation.
E. C. DAVIS, A. B., M. D., Obstetrics.
E. G. JONES, A. B., M. D., Gynecology.
R. T. DORSEY, Jr., B. S. M. D., Medicine.
L. M. GAINES, A. B., M. D., Internal Medicine.
J. N. LeCONTE, M. D., Disease of the Stomach and Intestines.
L. B. CLARKE, M. D., Pediatrics.
EDGAR PAULIN, M. D., Opsonic Medicine.
THEODORE TOEPEL, M. D., Mechano Therapy.
R. R. DALY, M. D., Medical Society.
A. W. STIRLING, M. D., etc., Diseases of the Eye, Ear, Nose and Throat.
BERNARD WOLFF, M. D., Diseases of the Skin.
E. G. BALLENGER, M. D., Diseases of the Genito-Urinary Organs.

VOL. LVIII. SEPTEMBER 1911 NO. 6.

### ETIOLOGY, PATHOLOGY AND TREATMENT OF PELLAGRA.

*(Pellagra and Oil Consuming Nations)*

Believing that the article in the August issue of this magazine contains sufficient evidence to show that cottonseed oil in unrestricted amounts incompatible with good health and that the consumption of cotton seed oil in the United States has been accompanied by the development and spread of Pellagra, I propose to offer the experience of the oil consuming nations. This information will show that all nations afflicted with Pellagra are oil consuming nations and that all nations which eat semi-drying oil are afflicted with Pellagra.

Reliable information along this line is somewhat disconnected. However, it shows a possible dietic and historical connection with Pellagra.

As maize has been given much prominence as an etiological factor in this disease, I have especially avoided any mention of the whole grain in this connection, preferring to confine what has been written to the oil.

I do not deny the possibility of eating enough maize to produce the disease. At the same time, it seems improbable, unless the diet excludes all other food, especially fats. It is also very probable that different degrees of diseased seed and grain will furnish an agent very potent in producing disease. Such a matter as rotten cotton seed or a few bushels of dead rats in all stages of putrefaction in a vat of oil might render refining inadequate.

It is probable that the oil from such seed (corn, cotton seed, poppy seed, sunflower, etc.), will produce much the same symptoms as are produced by the continuous eating of the sound oil, but with the difference that the symptoms from the diseased grain or seed would be immediate and transitory, while those from the sound oil would be delayed and in proportion to the quantity and length of time over which it is taken.

In order for any theory to merit credit it must be consistent with facts. The maize theory as it comes to us from French and Italian writers has failed to satisfy many scientific investigators because it requires too many inconsistencies to make it cover all the facts and incidents pertaining to the history of Pellagra. When the disease in an individual could not be traced to maize, the name of the disease is changed to "pseudo-pellagra." The supporters of the maize theory have had to inject some very far-fetched and contradictory arguments. They have had to claim that whiskey, corn starch and breakfast foods made from corn cause the disease.

We are told that it is a disease of poverty, yet we know a number of cases in America coming from people in the best of circumstances: that it is due to eating shipped corn, yet we see cases who select their own home raised grain and in others who eat no corn at all. To cover cases who eat no corn, they say that it is hereditary, yet on the next page they say it is not congenital, although it is often seen in infants.

We are asked to believe that it is not due to a living organism, or living virus, yet it reproduces itself and recurs year after year, even though corn has long since been excluded from the diet. On the other hand, cases are reported as recovering when spoiled corn was excluded from the diet.

Experiments with animals as carried out by Von Deckenback were accepted by Prof. Lombroso as evidence, even though it was shown that sound corn killed the animals.

There is much evidence in the report of experiments when subjected to careful analysis that point to the conclusion that the symptoms were produced by the oil or oxidized products of the oil. At the proper time and place I will attempt to show that the reported experiments tend to strengthen this idea.

It appears from questions asked me that I have perhaps not made clear what I conceive to be the agent or agents in these oils that are injurious. To further explain, I will state that this agent is linolin. It is a neutral fat which is present to some extent in all semi-drying and some drying oils.

When Linolin is consumed in large quantities it is deposited in the tissues as Linolin. When it undergoes oxidation poisonous products are formed. These oxidation products are suspected of producing the disease. This would necessarily mean that the disease is biochemical in nature. The amount of Linolin consumed will depend upon the per cent. present in the oil and the amount of oil eaten. Some of the semi-drying oils contain such a small per cent. of Linolin it is probable that they would not be deleterious to health. This point needs to be emphasized, as it appears that the quantity consumed is important.

In discussing this part ot the subject, we will use the table given in "Pellagra," by A. Marie, translated by Doctors Lavinder and Babcock, to show the geographical distribution of the disease. This list, given below, contains only those places from which the disease has been reported.

The data on oil consumption is in the most part obtained from reports from Special Agent Brode and Consul-General to the different countries. These reports are found in the Daily Consular and Trade Reports. Other information on fats and oils is obtained from Lewkowitsch on Fats and Oils. Much of the information relating to the history of Pellagra is also obtained from the above named work on Pellagra. After this explanation it is considered unnecessary to give any further specific reference.

### Geographical Distribution of Pellagra.

### *Endemic and Cases Relatively Numerous.*

Northern and Central Italy.
Roumania; Mouldavia, Wallachia.
Austria-Hungary; Tyrol.
Northern Spain.
Greece; Island of Corfu.
Lower Egypt and Red Sea Coast.

### *Endemic and Cases Relatively Few.*

Southwestern France.
North Portugal.
Austria-Hungary; Croatia, Dalmatia, Bosnia, Buckowina, Transylvania, Herzegovina, Galicia.
Servia.
Bulgaria.
Turkey.
Greece.
Russia; Bessarobia, Kherson, Poland.
Algeria.
Tunis.
Mexico; Yucatan, Campecpe.
South Africa; Kaffirs, Zulus, Robbin Island.
United States.
West Indes; Barbadoes, Jamaica.
Southern and Insular Italy.

*Sporadic.*

Asia Minor.
India; North Behar.
South America; Brazil, Argentina.
New Caledonia.
West Indes; Cuba, Porto Rico.

GEOGRAPHICAL DISTRIBUTION OF PELLAGRA IN THE UNITED STATES.

*Endemic and Cases Relaitvely Numerous.*

Virginia, North Carolina, South Carolina, Georgia, Florida, Alabama, Mississippi, Louisiana, Texas, Tennessee, Illinois.

*Endemic and Cases Relatively Few.*

Pennsylvania, Maryland, Kansas, Arkansas, Oklahoma, Kentucky, California.

*Sporadic or Doubtful.*

Massachusetts, Iowa, Ohio, New Mexico, Colorado, (?), Missouri, Vermont, Rhode Island, West Virginia, District of Columbia, New York (imported cases), New Jersey (imported cases), Indiana, Washington, and Michigan.

Take each region reporting Pellagra and study maize and oil producing seed industries. Prof. Lombroso states that Pellagra occurs in places that do not raise sufficient maize for home consumption, spoiled maize being imported. We shall see how well this statement is borne out. Keep in mind that the import or production of oil does not mean that the oil is always eaten. In a general way, it may be stated that the natives of all olive oil producing countries are oil eaters. Many nations import and some produce large quantities of oil of this class for commercial purposes. Germany is one of these. Germans are not an oil consuming people. The chief substitute for animal fat in Germany is a non-drying oil, viz., cocoanut butter, the daily production of which is estimated at one hundred tons. A law requires the use of ten per cent. of sesame oil in the manufacture of margarine. This amount I do not believe is sufficient to cause disease. It appears that it is necessary to introduce comparatively large amounts of linolin into the body in order to produce Pellagra.

Below is given a table of the semi-drying oils and nativity. Many minor oils are omitted, being less used and only supplementing the more common.

| Oil of | Nativity. |
| --- | --- |
| Cotton seed -- | United States, India, Egypt, China, Russia, Brizal, Mexico, Japan, Turkey, etc. |
| Sesame seed --- | The Levant, India Egypt, Java, Siam, Algeria, East and West Coast of Africa, So. Rhodesia. |
| Maize -------- | United States, Argentina, etc. |
| Beechnut ----- | Manufactured in Europe in 1713 but not at present. |
| Pinot --------- | Brazil and Guiana. |
| Kapok -------- | East and West Indies, South America, Mexico, Africa. |
| Brazil nut ---- | South America. |
| Luffa seed ---- | East India. |
| Rape seed ---- | India, Northern France. |
| Pumpkin seed - | Austria, Hungary, Russia. |
| Sunflower seed | Hungary, India, China, South and Southwest Russia. |
| Poppy seed --- | Asia Minor, Persia, India, Egypt, South Russia, Northern France. |

Poppy seed oil is a drying oil, but contains a large per cent. of Linolin and is an edible oil of extensive use. Some of the above oils contain a low per cent. of linolin and may be of no importance as an etiological factor.

Laws regulating the importation of seed oils into some olive growing countries have in recent years been restricted so as to protect the home industry. Some countries growing enormous quantities of oleaginous seed export the seed and consume very little or none of the oil. Such is the case in China and Japan.

### ITALY.

The people of Southern Europe are noted oil consumers. Italy is the second largest olive oil producing country in the world. Notwithstanding the enormous quantity of olive oil produced much oil is imported. They are also the largest exporters of comestible oils in this region.

Large amounts of semi-drying oils are imported. These are used at home and exported as edible oils under various labels and used to adulterate olive oil. These semi-drying oils are cheaper than olive oil, hence are consumed by the poor. Cotton seed oil was probably imported from Marseilles long before it was thought of in America. At the present time it is well known in the Italian market.

In recent years the demand for pure lard has increased. It is stated that when cultivation of lupines was introduced in certain regions, enabling the farming class to raise stock and dispense with maize as a food Pellagra disappeared. It is probable that instead of Pellagra disappearing for the above named reason it was in reality due to a change from vegetable oil to animal fat consumption.

### ROUMANIA: MOULDAVIA, WALLACHIA.

Special Agent Julian L. Brode states that much cotton seed oil (2,500 barrels) is consumed in Mouldavia, in the northern part of Roumania, but it is scarcely known to the consumer under its own name. The other principality named above is adjacent to this principality. Roumania raises sufficient corn for home consumption and some for export. Mr. Brode states that the total amount of oil consumed annually in Roumania is 25,000 barrels. About 20,000 barrels of olive oil and 2,500 barrels of cotton seed oil. There are no statistics at hand to show how long this proportion of oils has existed, or the former condition as to Pellagra and the kind of oils eaten in years gone by.

Here is another striking parallel in history. Note that Roumania raises sufficient corn and that Pellagra is reported from the only portion of the country in which the semi-drying oil is reported to be consumed. It is reasonable to suppose that some other oil has been imported in former years to make up the deficiency now supplied by cotton seed oil. As semi-drying oils are cheaper it is also reasonable to suppose that the imported oil was of this class.

### AUSTRIA-HUNGARY: TYROL.

There was a duty imposed on cotton seed oil in 1906 which limited importation of cotton seed oil. There is not sufficient olive oil produced in Austria-Hungary to supply home consumption of edible oils. The deficit, now that cotton seed oil is practically barred, is mostly supplied by sesame oil, arachis oil, sunflower seed oil, rape seed oil, imported olive oil and soja-bean oil. Some cotton seed oil, estimated at 2,500 to 3,000 tons, is crushed annually. In 1908 about 25,000 quintals of corn oil and 10,000 quintals of sunflower oil were imported. Pumpkin seed oil is a domestic product.

Doctors Lavinder and Babcocks translation furnishes the information that altogether a population of 2,250,000 has been investigated since 1905, with the result that 78,163 persons (three per cent.) were found to be suffering from the disease. Statistics suggest that Austria-Hungary produces sufficient maize for home consumption.

### NORTHERN SPAIN.

Until the law of July 5, 1892, regulating the importation of seed oils, Spain imported large quantities of cotton seed, poppy and sesame oil, also other oils of this class. They were consumed by the natives and used to adulterate olive oil as it is at present done in France and Italy.

The law of 1892 provided a means for rendering them useless, with the object of preventing the adulteration of Spanish olive oil, whether destined for home consumption or exportation.

The following remarks from The Epoca, January 9, 1909, are particularly significant: "Almost daily complaints are heard that in Northern Spain olive oil is being mixed with oleaginous substances contrary to the law." Spain produces enough olive oil for home consumption and for export trade to France and Italy. The quantity exported is often so large that the natives are forced by high prices and scarcity to eat imported semi-drying oils."

### GREECE: ISLAND OF CORFU.

Greece is a large consumer of edible (olive) oil and except in short-crop years, which happen almost every other year, export trade suffers. Only about ten per cent. of the oil is strictly edible. This comes from Corfu and Paxi and is exported to France and Italy.

The appearance of Pellagra in Corfu has been of recent date, twenty years ago. No information is at hand to show the quantity of oil imported into Greece before and during this time. However, there is evidence that before March, 1909, at which time the duty was raised, cotton seed oil was imported.

It was stated that concident with the reduction of corn growing and the increase in importation of corn as the result of extensive vine growing, there was development of Pellagra. It would be interesting to know if the extension of vine growing had the same effect upon olive oil production that it had upon corn production. If so, the importation of oil was necessary.

### LOWER EGYPT AND RED SEA COAST.

The natives, most of whom are Mohammedans and large oil consumers, have been educated to substitute cotton seed oil for olive oil (or adulterated olive oil) they formerly used, and the latter is now found only in the homes of the wealthy.

The report of Pellagra in Egypt and the Red Sea Coast is of recent date and is well within the period of the introduction of cotton seed oil. This change is now taking place to a great extent in Turkey. In 1907 there were seven cotton oil mills in operation in Egypt. Sesame seed and poppy seed are indigenous.

### SOUTHWESTERN FRANCE.

We have in this country a striking parallel, as forcible and convincing as the history of cotton seed products and Pellagra in the United States. Marseilles is the leading vegetable oil center of the world. The first mill for crushing seed oil was started in 1817. There are now forty-five mills crushing annually about 465,000 tons of seed. In Marseilles cotton seed were being crushed when they were being thrown away in America.

Peanuts, sesame, copra, linseed, colza, and poppy seeds are crushed. Most of the cotton seed oil is mixed with other oils and sold as "table oil." At the present time olive oil or peanut oil, and sesame oil are the oils most used in competition with cotton seed oil. Olive oil is in a class by itself. Good edible oils, sold at the same price as cotton seed oil, are preferred to the latter in the majority of cases.

Poppy seed oil is brought into commerce from France both in the south (Marseilles) and in the north, and in Germany. The best qualities of oil are used for edible purposes, also for fine paints. Other uses are for white pigments and in the manufacture of wax oil and soap making. France crushes about 40,000,000 kilograms of poppy seed annually and in 1903 imported 123,239,000 kilograms of sesame seed. Oil from fermented or over-heated sesame seed has been shown to be poisonous. The oil cake from such seed is poisonous to cattle.

Note that the above named oils relate to the oil consumption of the present day. The oil first produced in France was olive oil; then poppy seed oil in large amounts in 1817. There seems to have been a marked change in the kind of oil eaten in recent years. Oil of poppy, because of the larger per cent. of linolin contained, is suspected to be more injurious than oil of sesame. I am also led to believe that "vegetaline" is at present consumed in large amounts among those who prefer vegetable fat to animal fat. Vegetaline is made chiefly from an extract of cocoa bean and is harmless.

Bearing in mind that corn had been eaten in France for years and the date of the introduction of seed oil on a large scale into this country, review the history of Pellagra. The first communications to the Royal Society of Medicine on Pellagra were in 1829. Consider the introduction of oil eating into America and the fact that we have for centuries eaten corn, and spoiled corn at times.

There are two changes that have taken place in France to account for the decrease of Pellagra in some sections. About the time the decrease began the landed estates were divided and the condition of the peasantry thereby much improved. The other change is suggested above, as being a change from semi-drying oil to non-drying oil. Quite significant in connection with the region now afflicted by Pellagra is the fact that at the present time a low grade of olive oil, made chiefly of cotton seed oil shipped from the United States, is largely used in the kitchens of the southern portion of France, lard and butter being too expensive.

### NORTH PORTUGAL.

Portugal at present prohibits the impotration of comestible cotton seed oil. The annual production of olive oil is about 30,000 tons, which is not enough to meet domestic demands. Other oils are imported to meet the requirements of the people.

### SERVIA.

According to Consul Robert S. S. Bergh, Belgarde, cotton seed oil imported from the United States and England has become a strong competitor to olive oil, under which name it is often sold at a handsome profit. He says that this alone is the best guarantee that cotton seed oil will gradually replace the olive oil and sesame oil, the imports of which are still considerable. There is no Servian cotton seed oil to compete against. as the only Servian oil factory was removed to Salonica, Turkey.

We have here a striking incident when the import of cotton seed oil is considered in connection with the increase in Pellagra. According to Servian custom statistics, there were no imports from the United States in 1907; 350 barrels in 1908; 655 barrels in 1909; 3,700 barrels in 1910; and 6,000 barrels expected in 1911. Prior to the above statistics most of the oil came from England. It appears that the above may bear a relationship with the four-fold increase in Pellagra.

### BULGARIA.

There is practically no comestible oil produced in Bulgaria. No cotton seed oil has been imported in the last seven years except denatured oil. There are imported annually about 18,000 barrels of comestible oils, composed mostly of olive oil, sunflower seed oil, sesame oil, and arachis oil, amounts being in the order named.

Pellagra is on the increase in Bulgaria.

### TURKEY, IN EUROPE.

Turkey in Asia produces sufficient olive oil for home consumption and until 1907 prohibited the importation of edible products. So far no Pellagra has been reported.

In some olive growing countries the use of other than vegetable oils is greatly restricted, or in many cases prohibited, by religious laws and customs. For this reason there is increased demand for edible oils. Since 1907 cotton seed oil has been used in competition with oil of sesame and other similar oil for mixing with olive oil. Recent increase of Pellagra in European Turkey is reported. Cotton seed is crushed for oil at Salonica.

### RUSSIA.

The provinces named as having reported Pellagra are in Southern and Southwestern Russia.

Two kinds of sunflower seed are raised. One is used as a food product and not crushed. It is a very common sight to see Russian peasants walking along intently absorbed in eating sunflower seed. The variety raised for crushing comprises only about twenty-five per cent. of the entire crop. The oil produced in 1908 was 576,000 tons. Much cotton seed oil is produced and consumed in Russia. It is significant that the sunflower is grown in the region from which Pellagra has been reported. Pumpkin seed oil is used to some extent for edible purposes. This region produces sufficient corn for home consumption.

### ALGERIA: TUNIS.

These states are inhabited by Berbers, a pastoral and agricultural people. The other principal races are Moors, Arabs, Turks from Asia, and French from Europe, all but the last named being Mohammedans.

Tunis being a dependency of Turkey and Algeria a province of France, the same conditions in regard to the subject under discussion probably obtain as exist in the sovereign country.

Sesame seed is indigenous to Algeria.

### CUBA.

The conditions in Cuba favor the development of Pellagra from the maize standpoint because of the fact that two crops of corn are produced annually, yet Pellagra is not frequent and probably within the cotton seed oil period.

### MEXICO.

Cotton seed is crushed for oil in Mexico. In 1907 there were seven cotton oil mills in operation.

Oil of kopok is a domestic product and has been used for edible purposes for years.

Pellagra was first reported between 1882 and 1891.

### NEW CALEDONIA.

New Caledonia belongs to France and it is reasonable to suppose that the conditions are somewhat the same here as in France. The nature of the inhabitants does not call for a high class of food.

### PORTO RICO.

Information is not at hand to show use of semi-drying oils, however, Porto Rico is in the region where oil seeds abound.

### S OUTH AFRICA.

This region produces sufficient maize for home consumption. Oil of kopok s native to Africa and is consumed.

In 1895 South Africa imported 122,146 gallons of cotton seed oil from United States alone.

### WEST INDIES.

Oil of kopok has been used for edible purposes tor years. Many other oil seeds are indigenous to these islands and the other island in which Pellagra is sporadic.

### UNITED STATES.

The classification of this country among the relatively few was probably correct when the translation by Drs. Babcock and Lavinder was written, but they would no doubt now change it to relatively numerous. Notwithstanding there has been a general warning sent out for the past four or five years against the eating of spoiled corn, there has been a rapid increase in the development of Pellagra. Many who have been most careful in the selection of their corn products have fallen victims. There is no geographical distribution in the Southern States. All ages and either sex are afflicted alike. Two hundred cases show a percentage of sixty-two in women. Two children, five and seven years, are among the number. At the same ratio of increase there will be an appalling number of Pellagrins in the South in 1912.

For the comparative increase in the consumption of cotton seed oil I submit the figures given in the August issue of this magazine. These figures show that only ten per cent. of the white people were eating cotton seed oil three years ago. This has increased to fifty per cent. at the present time. In 1897 there were retained for home consumption 37,923,118 gallons; in 1907 this increased to 133,844,536 gallons.

To show that the shipping of corn from one point to another has not in every case been necessary is given below the corn crop of the various states in 1907.

PRODUCTION OF CORN IN THE UNITED STATES, 1908, BY STATES.

| *State or Territory.* | *Bushels.* |
|---|---|
| Maine | 567,000 |
| New Hampshire | 1,092,000 |
| Vermont | 2,499,000 |
| Massachusetts | 1,818,000 |
| Rhode Island | 428,000 |
| Connecticut | 2,395,000 |
| New York | 24,250,000 |
| New Jersey | 10,564,000 |
| Pennsylvania | 57,275,000 |
| Delaware | 6,240,000 |
| Maryland | 24,705,000 |
| Virginia | 50,050,00 |
| West Virginia | 23,962,000 |
| North Carolina | 50,166,000 |
| South Carolina | 29,299,000 |

| | |
|---|---|
| Georgia | 53,750,000 |
| Florida | 6,584,000 |
| Ohio | 136,675,000 |
| Indiana | 137,835,000 |
| Illinois | 298,620,000 |
| Michigan | 60,420,000 |
| Wisconsin | 49,674,000 |
| Minnesota | 46,835,000 |
| Iowa | 287,456,000 |
| Missouri | 203,634,000 |
| North Dakota | 3,856,000 |
| South Dakota | 57,677,000 |
| Nebraska | 205,767,000 |
| Kansas | 156,200,000 |
| Kentucky | 84,823,000 |
| Tennessee | 83,080,000 |
| Alabama | 44,835,000 |
| Mississippi | 45,845,000 |
| Louisiana | 33,898,000 |
| Texas | 201,848,000 |
| Oklahoma | 122,239,000 |
| Arkansas | 54,035,000 |
| Montana | 94,000 |
| Wyoming | 84,0000 |
| Colorado | 2,586,000 |
| New Mexico | 1,755,000 |
| Arizona | 432,000,000 |
| Utah | 323,000 |
| Idaho | 174,000 |
| Washington | 332,000 |
| Oregon | 445,000 |
| California | 1,600,000 |
| Total | 2,668,651,000 |

From the Year Book, U. S. Department of Agriculture, 1908, pages 599 and 603.

The exports of oil of corn from the United States amounted to about 4,000,000 gallons annually.

Corn crop of the various countries:

CORN CROP OF COUNTRIES NAMED, 1907.

| NORTH AMERICA: | BUSHELS. | BUSHELS. |
|---|---|---|
| United States | | 2,592,320,000 |
| Canada: | | |
| Ontario | 22,949,000 | |
| Quebec | 1,420,000 | |
| Mexco | | 70,000,000 |
| SOUTH AMERICA: | | |
| Argentina | | 71,768,000 |
| Chile | | 1,500,000 |
| Uruguay | | 5,359,000 |
| EUROPE: | | |
| Austria-Hungary: | | |
| Austria | 16,599,000 | |
| Hungary proper | 155,616,000 | |
| Croatia-Slavonia | 17,934,000 | |
| Bosnia-Herzegovina | 6,468,000 | |
| Bulgaria | | 12,000,000 |
| France | | 24,027,000 |
| Italy | | 88,428,000 |
| Portugal | | 9,000,000 |
| Roumania | | 57,576,000 |
| Russia | | |
| Russia proper | 41,903,000 | |
| Poland | 1,000 | |
| Northern Caucasia | 8,860,000 | |
| Servia | | 17,691,000 |
| Spain | | 25,372,000 |
| AFRICA: | | |
| Algeria | | 402,000 |
| Cape of Good Hope | | 3,550,000 |
| Egypt | | 35,000,000 |
| Natal | | 3,300,000 |

| | | | |
|---|---|---|---|
| Sudan (Anglo-Egyptian) ----------- | | 300,000 | |
| AUSTRALASIA: | | | |
| Australia: | | | |
| Queensland ------ | 3,820,000 | | |
| New South Wales - | 5,945,000 | | |
| Victoria --------- | 727,000 | | |
| Western Australia --- | 1,000 | | |
| New Zealand ---- | | 419,000 | |
| Grand Total ------- | | | 3,300,255,000 |

From the Year Book, U. S. Department of Agriculture, 1908, page 597.

This is an incomplete statement, as corn, like other oil producing seed, is cultivated in almost every country where the climate is adapted to its growth. It appears that the consumption of semi-drying oils has been slower in developing than the introduction of maize as a food. Also it appears that the spread of Pellagra has been more slow than the progress of corn consumption, and seems to follow and parallel the more slow process of oil-eating education.

My conclusion from investigations of the seed oil industry is that since 1817, when the first seed crushing mill was put into operation in Marseilles, there has been almst an unlimited supply of seed oils. The habits of the various nations and individuals have alone operated in determining the extent of oil consumption. Often the need of cheap food has determined the selection. Until the mills began crushing seed there was no Pellagra in France. The peasantry were afflicted mostly because the poor buy cheap food. In the United States the selection of edible fat was not determined by price until the cost of provisions increased, about 1908. Until this date, purity of food was the determining factor to a great extent. It is true that the manufacturers have appealed to patronage from both standpoints. They made a cheap article for the poor and a high-priced article for those in better circumstances. Dyspeptics have been shown that the oil is more digestible. The fastidious are told that the oil is pure vegetable oil, clean and highly nutritious. The Pure Food and Drug Act is stamped upon each package as a guarantee of purity and wholesomeness. The unsuspecting public, depending upon the guardianship of the Government stamp, has adopted cotton seed oil as a regular article of diet. Various cooking substances, without a single indication of their nature, are coming into the market without even the distributing agent being able (or willing) to name the contents of the package. People are consuming these preparations without question because the Government stamp is impressed upon them. This being the case in our own country, who will doubt that the cheap comestible oils shipped (even as pure olive oil) into oil consuming communities are made up largely of semi-drying oil?

Note the progress of Pellagra in the United States. It started in the South, where oil consumption began, and only after it began. Illinois should be placed with the South both as to oil consumption and Pellagra. More Pellagra has appeared in California than any other Western State. It is significant that she also consumes more cotton seed oil. A map of cotton seed oil consumpton in the United States would serve as a map showing the geographical distribution of Pellagra, except as affected by climate. Nations that have remained consumers of non-drying oils are not afflicted with Pellagra. Some of them have eaten maize just as the inhabitants of the United States have for several centuries without having developed Pellagra.

Many investigators have probably failed to establish the eating of cotton seed oil products by their cases. The difficulty in getting a correct history has often been very discouraging. The facts were gotten in some cases with much labor and expense. Cases have been seen who have eaten a lard substitute for years under the impression that it was pure leaf lard. Physicians and others have pointed out Pellagrins who, they claimed, had never eaten any fat except pure lard. Investigation of over two hundred consecutive cases of Pellagra showed that every one of them had eaten cotton seed oil more than eight months.

One patient said on four different days that he raised his own hogs and rendered his own lard. In a few days his wife came to Atlanta and on three different days repeated his statement. On the next inquiry I changed the form of my question and received the reply that they had quit farming eighteen months ago and gone into the grocery business. During this time they had bought leaf lard. Complying with my request, they wrote home and found they had been eating a popular brand of lard substitute that bore a label stating that it contained no hog lard. (This case came near stopping my investigations.)

Experiments upon animals indicate that Pellagra may be caused by eating less than one ounce of oil daily. If this is true, oil may be consumed for medicinal purposes in sufficient quantity to produce the disease, and olive oil should not be administered unless of known quality. Some idea is gained of the danger by two labels reproduced below.

Fig. 1 Lable on Bottle of Maize Oil

Fig. 2 Lable on Bottle of Cotton seed Oil

Reproduced from Bulletin 77, Bureau of Chemistry.

The table given below is obtained from the same Bulletin:

Serial No. 21027—Huile d'Olive Vierge. E. Loubon, Nice France.

Serial No. 21031—Olio d'Oliva. Egisto Dini, Lacca, Italy.

Serial No. 21110. Sublime Olive Oil. Addisoni Fils, Masina, Italy.

Serial No. 21113.—Huile d'Olive, Extra Surfine. Jules Chambon & Cie., Bordeaux, France. Imported by Chas. W. Longaker, Pottstown.

Serial No. 21395.—Huile d'Olive. P. M. Loubric, Bordeaux, France. Packed for I. K. Bean.

Serial No. 21396.—Huile d'Olive. P. M. Loubric, Bordeaux, France. Imported for J. M. Oliver & Sons.

Serial No. 21906.—Lucca, Italy.

Serial No. 22924.—Olio Sopraffino. Umberto Albertini, Livorno, Italy.

Serial No. 22925.—Olio Puro d'Oliva. Restivo & Co., Lucca, (Tuscana) Italy.

Serial No 23070.—Huile d'Olive, Vierge. Silas Peirce & Co., Bordeaux, France.

Serial No. 23072—Huile d'Olive, Vierge, d'Aix. Dupont & Cie, Bordeaux, France.

Serial No. 23085.—Huile d'Olive, Extra Vierge. Naegely & Pasero, Marseille, France.

Serial No. 83086—Huile d'Olive, Vierge, d'Aix. Alex. Euquem, Bordeaux, France.

Serial No. 23087.— Olio d'Oliva. Luigi di Cos. Matteucci, Lucca, Italy.

Serial No. 23090.—Olio d'Oliva, Sopraffino. F. Berio & Co.. Lucca, Tuscana, Italy.

Serial No 23096—.Huile d'Olive, Extra Surfine. Tisserand Fils, Bordeaux, France.

Serial No. 23108.—Olio d'Oliva, Sopraffino. Gaetano Guirlani, Lucca, Italy.

Serial No. 23120.—Olio d'Oliva Vergine. De Martini E. Cia., Rivera di Genova, Italy.

Serial No. 23121.—Olio Vergine Purissimo. Garantito di Lucca, S. Scatena & Co.

Serial No. 23131.—Huile d'Olive, Clarifee. Martinot Freres, Bordeaux, France.

Serial No. 23089—Huile Salad. Giacomo Luigi, Nove Orleons, Americano.

Serial No. 21030.—T. A. Fueich & Son, Lussinpiccolo, Austria

Serial No. 22916.—Huile d'Olive. Frerres & Du Peaux, Bordeaux, France.

Serial No. 22917.—Superior in Quality, Purity, and Flavor to any Olive Oil in the market. Dove Pure Oil Co.

Serial No. 23098.—Olive Oil. El Montecito Man'f'g. Co., El Montecito, Cal.

Serial No. 23102.—"Ramona" Pure Olive Oil. W. P. Wheeler, Oakland, Cal.

Most of these oils were bought in open market for pure olive and found to contain oil of maize, oil of sesame, and oil of cotton seed. The others were obtained from the Appraiser's office.

Some idea of the various uses of cotton seed products as a human food may be obtained from the following table:

| | | | |
|---|---|---|---|
| Cotton Seed | Oil Cake and Meal | Confectionery. | |
| | | Flour | Bread.<br>Cake.<br>Cracker. |
| Summer Yellow Oil | Winter Yellow Oil. | | |
| | Summer White Oil. | Lard Compound.<br>White Cotolene. | |
| | Stearin | Butter Oil.<br>Cooking Oil<br>Salad Oil<br>Olive Oil (so-called).<br>Oleomargarine. | |
| | Miscellaneons | Setting Olives.<br>Packing Sardines.<br>Emulsion for Medicinal Purposes | |

From Bulletin 111, the Department of Commerce and Labor, Bureau of the Census.

401 Empire Bldg.

## Figures and Tables

**Fig. 1 f1:**
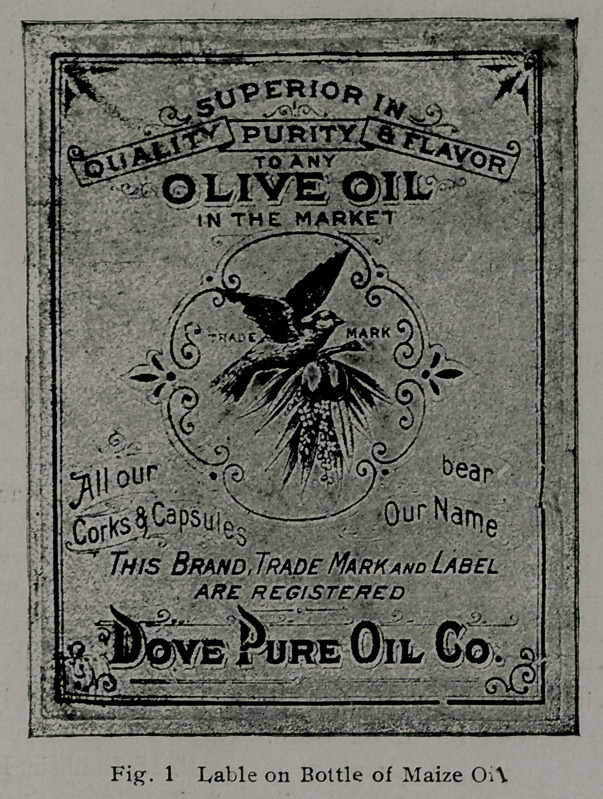


**Fig. 2 f2:**
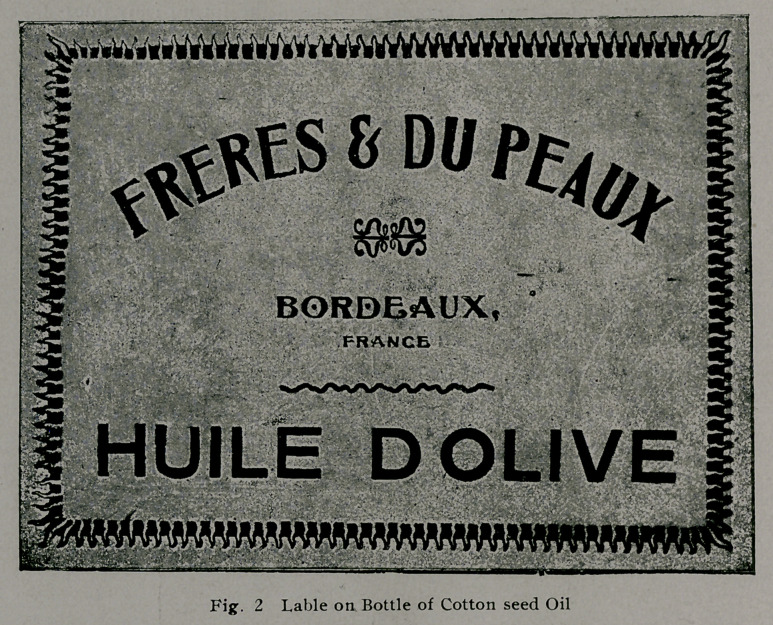


**Figure f3:**